# Deep and Prolonged Response to Aurora A Kinase Inhibitor and Subsequently to Nivolumab in MYCL1-Driven Small-Cell Lung Cancer: Case Report and Literature Review

**DOI:** 10.1155/2020/8026849

**Published:** 2020-04-06

**Authors:** Bhaskar C. Kolla, Emilian Racila, Manish R. Patel

**Affiliations:** ^1^Division of Hematology and Oncology, University of Minnesota, 420 Delaware St SE, MMC 480, Minneapolis, MN 55455, USA; ^2^Division of Laboratory Medicine and Pathology, University of Minnesota, 420 Delaware St SE, MMC 76, Minneapolis, MN 55455, USA

## Abstract

Small-cell lung carcinoma (SCLC) is one of the most aggressive solid tumors, and the prognosis has not improved significantly in 25 years. Despite a recent understanding of the genomic aberrations seen in SCLC, these insights have not led to any breakthroughs in treatment. We present a patient with SCLC harboring a novel MYCL1 fusion protein who experienced a prolonged disease course due to the use of Aurora A kinase inhibitor and subsequently nivolumab. MYC family genes are master regulators of several cellular pathways including proliferation, differentiation, and apoptosis and recently have been shown to be involved in tumor immune evasion. Large studies have shown that a significant proportion of patients with SCLC have amplification or overexpression of MYC family genes. Preclinical data have exposed vulnerability of MYC-driven tumors to Aurora kinase inhibitors, bromodomain and extraterminal domain inhibitors, and recently to immune checkpoint blockers. Further studies using these agents with selective enrolling of patients with MYC-altered tumors are warranted to exploit these vulnerabilities.

## 1. Introduction

Small-cell lung cancer (SCLC) accounts for approximately 13% or 29,000 of all lung cancers annually in the United States [1]. The vast majority of these patients are current or former smokers. SCLC is characterized by a high proliferation rate, rapid doubling time, and early development of distant metastases [2]. As a result, approximately 70 percent of patients present with overt metastatic disease. Limited stage (LS) disease, defined as tumor confined to one radiation field, is potentially curable with combination chemotherapy and radiation, but most patients will eventually relapse with distant disease and ultimately succumb to the disease. Even in patients who present with extensive stage disease (defined as disease spread beyond one radiation field), SCLC is almost uniformly responsive to initial chemotherapy and radiation therapy; however, early relapse is common. Beyond first line therapy, several agents have shown activity, but response rates are typically less than 20%. Median survival is approximately 23 months in limited stage disease and 12 months in extensive stage disease [2, 3]. There have been many clinical trials in SCLC in the past 25 years without significant improvement in clinical outcomes [4]. Genomic studies of SCLC have identified several alterations, such as genes in MYC and mTOR pathways, which are potentially druggable [5–8]. Clinical trials targeting mTOR and MYC pathways have been disappointing [9, 10]. These trials included patients without consideration of the tumor molecular profile, which may in part explain the lack of promising results. Other reasons cited for lack of progress in SCLC are the limited availability of tissue for analysis, molecular complexity, and the high mutation burden [4].

We present here a patient with an unusual case of SCLC who was found to have MYCL1 fusion, with deep and prolonged response to Aurora kinase inhibitor (AKI) and then to immune checkpoint blockade. We discuss possible mechanisms that would explain this response and a review of the literature regarding such responses. An informed consent was obtained from the patient.

## 2. Case Presentation

**Figure 1:**
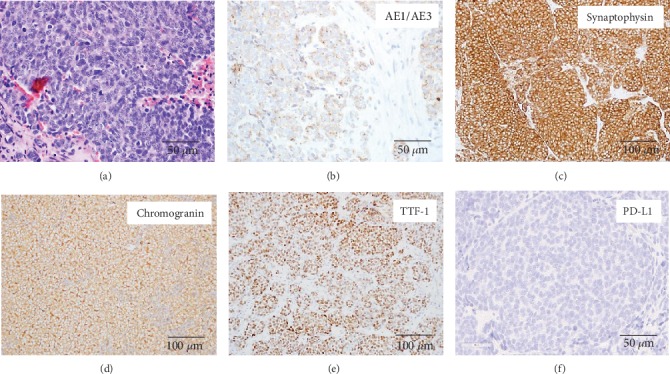
Morphologic features and immunophenotype of SCLC from supraclavicular lymph node biopsy obtained at the time of diagnosis in 2009. (a) H&E stain displaying the characteristic morphologic features of small-cell carcinoma including high nuclear to cytoplasm ratio, hyperchromatic nuclei with salt and pepper chromatin, inconspicuous nucleoli, and frequent mitoses. (b) AE1/AE3 stain demonstrating focal punctate or discontinuous staining that is usually observed in SCLC. (c, d) The neoplasm is diffusely positive for neuroendocrine markers synaptophysin and chromogranin. (e) TTF-1 expression is positive, suggesting pulmonary origin of tumor. (f) PD-L1 immunostain was negative in the initial biopsy and subsequent biopsied metastatic sites.

A 46-year old nonsmoker male presented in December of 2007 with right supraclavicular lymphadenopathy. An excisional biopsy of the lymph node was performed. Histopathology (Figure 1) showed the morphologic features of SCLC including small to medium size cells, high nuclear/cytoplasmic ratio, salt and pepper chromatin with inconspicuous nucleoli, nuclear molding, and high mitotic activity. Immunostaining showed that the tumor cells expressed synaptophysin and chromogranin and discontinuous cytokeratin markers. TTF-1 was also positive. Imaging was performed with PET/CT showing a 5 cm right hilar mass and right paratracheal lymphadenopathy and no disease elsewhere including a negative brain MRI.

**Figure 2:**
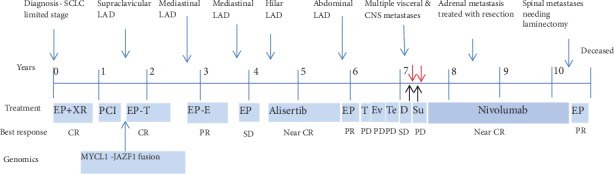
Patient's disease course timeline. Black arrow: Surgical resection of intramedullary metastasis at C6-7; Red arrow: stereotactic radiosurgery to 6, 9, and 11 brain lesions; SCLC: small-cell lung cancer; CR: complete response; PR: partial response; SD: stable disease; PD: progressive disease; EP: etoposide/platinum; T: topotecan; PCI: prophylactic cranial irradiation; E: etoposide; Ev: everolimus; Tem: temozolamide; D: docetaxel; Su: sunitinib; LAD: lymphadenopathy; XRT: external beam radiation therapy.

Thus, he was deemed to have limited stage disease and was treated accordingly with cisplatin and etoposide and concurrent radiation therapy (Figure 2). He achieved a complete response after 6 cycles of chemotherapy and subsequently underwent prophylactic cranial irradiation. He was monitored clinically and by imaging every 3 months. In May 2009, the disease relapsed with left supraclavicular lymphadenopathy and was confirmed by excisional biopsy. He underwent radiation therapy with concurrent cisplatin and etoposide for 2 cycles followed by 4 cycles of oral topotecan. He had complete response again that lasted for one year. In September 2010, he had a relapse presenting with mediastinal lymphadenopathy. After another 5 cycles of cisplatin and etoposide, he had near complete response, and he was monitored clinically. After progression in September 2011 with increased hypermetabolic activity in the right hilum and paratracheal lymph nodes, he was started on carboplatin and etoposide. He had good response after 4 cycles and was switched to oral etoposide. He again progressed with increased metabolic activity in the right hilar and paratracheal region.

**Figure 3:**
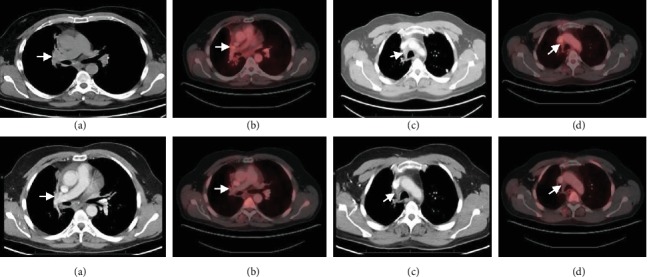
Top—CT and PET-CT images April 2012 before alisertib showing 2.1 cm R hilar mass (a) with SUV 4.8 (b) and R para-tracheal soft tissue prominence with fibrotic changes in previously irradiated field (c) with SUV 5.2 (d). Bottom—CT and PET-CT images December 2012 after 10 cycles of alisertib showing resolution of R hilar mass (a, b) and unchanged R para-tracheal cicatricial changes (c) with maximum SUV of 2.3 (d).

At this time, genomic profiling of his prior tumor biopsy was undertaken. This showed that his tumor harbored a novel JAZF1-MYCL1 gene fusion but lacked alterations in TP53 and RB1. This was performed in a CLIA-certified, CAP-accredited commercial laboratory. The technique used was next-generation sequencing with hybridization-captured, adaptor ligation-based libraries to high, uniform coverage (>500×) for all coding exons for 236 cancer-related genes plus 46 introns from 19 genes frequently rearranged in cancer [11]. All classes of genomic alterations (GA) were identified including base substitutions, insertions/deletions, copy number alterations, and rearrangements. Although mutations in TP53 and RB1 are observed in vast majority of SCLC cases, a small fraction of these tumors can be wild type for TP53 and RB1 [5]. There were no mutations in EGFR, BRAF, and MET or rearrangements in ALK or ROS1 that are seen in other types of lung cancer. He was then enrolled in a clinical trial with Aurora A kinase inhibitor (MLN8237/Alisertib-50 mg BID for 7 days of 21 days cycle), in April 2012 [6, 12]. He experienced an objective response after 4 cycles and near complete response after 10 cycles of therapy (Figure 3). He remained on this drug for 23 cycles (18 months). In September 2013, he developed progression with aortocaval lymphadenopathy. Over the next 18 months, he was treated with several chemotherapeutic agents with disease progression as his best response (carboplatin plus etoposide, topotecan, everolimus, temozolamide, docetaxel, and sunitinib). His disease progressed to involve several organs including the brain, spinal cord, liver, pancreas, adrenals, bone, and pleural, pericardial, and peritoneal spaces. During this time, he underwent several palliative procedures including spinal decompression surgery, multiple instances of stereotactic brain radiation, and ureteral and biliary stents to relieve obstruction.

**Figure 4:**
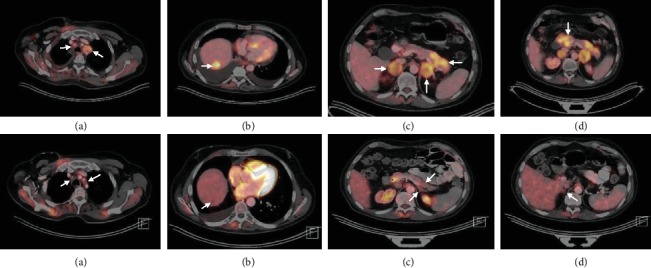
Top—PET-CT chest, abdomen, and pelvis before initiation of nivolumab (July 2015) showing R para-tracheal soft tissue thickening and 3.2 cm L para-tracheal lymph node (a), 2.6 cm R hepatic dome lesion (b), 3.8 cm R adrenal and 4.1 cm L adrenal nodules (c), and multiple pancreatic lesions in the head, body, and tail (c, d). In addition (not shown), the patient had other metastases involving bones, mediastinal and retroperitoneal lymph nodes, and pleural and peritoneal surfaces. The maximum SUV values for R-para-tracheal thickening, L para-tracheal lymph node, hepatic lesion, R adrenal nodule, L adrenal nodule, and pancreatic lesions were 5.2, 6.83, 13.08, 11.9, 9.9, and 11.4, respectively. Bottom—PET-CT chest, abdomen, and pelvis after 16 weeks of nivolumab (December 2015) showing resolution of L para-tracheal lymphadenopathy (a), decrease in R hepatic dome lesion to 1.2 cm (b), decrease in L adrenal nodule to 1.4 cm (c), and resolution of R adrenal (d), and all pancreatic lesions (c, d). Other metastatic lesions (not shown) in the bone, mediastinal and retroperitoneal lymph nodes, and pleural and peritoneal surfaces have completely resolved. The maximum SUV for R hepatic dome lesion has reduced from 13.08 to 2.0, and no increased metabolic activity was detected in the L adrenal nodule. Other than equivocal increased metabolic activity in previously irradiated R para-tracheal region (SUV 2.4), there was no increase in metabolic activity anywhere else.

He was then started on nivolumab (3 mg/kg every 2 weeks) in August 2015 based on preliminary results from a phase I/II study [13]. He had a dramatic response. Initially, he had recurrent pleural effusions requiring 6 thoracenteses in the first few weeks as well as pericardial effusion with cardiac tamponade requiring pericardial window [14]. He had evidence of partial response at 8 weeks of therapy and near complete response at 16 weeks of therapy in December 2015 (Figure 4). He developed local recurrence in right adrenal gland in May 2016, which was treated by right adrenal gland resection. Unfortunately, the disease progressed again in April 2018 with transient response to platinum doublet. This was followed by a rapid progression, and he succumbed to disease in October 2018.

## 3. Discussion

This report describes a patient with unusual SCLC with a very long disease course and deep response to AKI and remarkable response to immune checkpoint blockade. There are several unique aspects to this case that are noteworthy. Firstly, this patient was a never smoker. SCLC occurring in nonsmokers has been previously reported [15]. More recently, SCLC has been seen in the de-evolution of EGFR-driven NSCLC [16]. Our patient did not have EGFR mutations at any time in his disease course. Secondly, the lack of TP53 or RB1 alterations is also unique. Though TP53 and RB1 alterations are reported in >90% of SCLC cases, this is not universal [5]. The morphologic features over several biopsies taken during his disease course, clinical sites of presentation, and metastatic disease course were all consistent with SCLC; thus, it was difficult to classify this tumor into a different entity. Finally, the tumor harbored a unique JAZF1-MYCL1 fusion. Activation of MYC family of genes has been shown to sensitize tumors to Aurora kinase inhibitors [17]. MYC, MYCN, and MYCL are three versions of a family of genes that regulate multiple activities involving cell cycle, growth, metabolism, differentiation, apoptosis, transformation, and immune-regulation [18, 19]. Although JAZF1-MYCL1 fusion has never been characterized before, a similar fusion involving RLF-MYCL1 has been well characterized in SCLC samples [20, 21]. Using RNA-seq and RT-PCR analyses, Rudin et al. showed that RLF-MYCL1 fusion is a recurrent event in SCLC that leads to production of a fusion protein [20]. Small interfering RNA targeting of MYCL1 led to significant reduction in proliferation of these cell lines. Iwakawa et al. performed copy number and whole transcriptome analysis on 58 and 42 SCLC samples, respectively [21]. They found frequent amplification of MYCL1, MYC (6/58), and MYCN (2/58) genes. A study of 689 cases of SCLC assayed by hybrid-capture-based comprehensive genomic profiling identified 53 cases of MYCL1 amplification including 6 cases of MYCL1 fusion, all 6 of them with a different fusion partner [22]. Sos et al. showed that short hairpin RNA (shRNA) knockdown of MYC, Aurora kinase (AURK) genes, or use of small molecule AKIs resulted in apoptosis of MYC-amplified cell lines but not nonamplified cell lines [6]. Recent work in a mouse model has faithfully replicated the sensitivity of MYC-driven SCLC to AKI in combination with chemotherapy [17]. Similarly, in another preclinical study using SCLC cell lines as well as xenograft models, activation or amplification of any of the three MYC family genes strongly predicted response to Aurora kinase inhibitor PF-03814735 [23]. Aurora kinase A (AURKA) forms a complex with and stabilizes N-Myc in MYCN-driven neuroblastoma cells, and small molecule AURKA inhibitors have been shown to promote degradation of N-Myc. In a murine model of MYCN-driven neuroblastoma, AURKA inhibitors have been shown to induce tumor regression and prolong survival [24]. A very similar effect was also seen in MYC expressing human hepatocellular carcinoma cell lines and xenograft model [25]. In a phase II study of Paclitaxel plus Alisertib vs. placebo, longer PFS was achieved in c-Myc-positive patients treated with alisertib/paclitaxel than in patients given placebo/paclitaxel (4.64 vs. 2.27 months; HR, 0.29). Conversely, patients who were c-Myc negative had a shorter PFS with the combination (3.32 vs. 5.16 months; HR, 11.8) [10]. In our patient, the presence of JAZF1-MYCL1 fusion without additional genomic alterations may have made it particularly sensitive to AKI, though this is speculative.

How is this MYCL1 activation related to sensitivity to PD-1 blockade? The answer undoubtedly requires focused laboratory investigation. This patient had no expression of PD-L1 on neoplastic cells, both at diagnosis and on tissue specimen obtained in 2015 prior to treatment with nivolumab. PD-L1 expression correlates with response to PD-1 blockers, but it is not a requirement for activity of these drugs. There have been several preclinical studies that implicate Myc in tumor immune-evasion, and Myc amplification may sensitize tumors to immune-checkpoint blockade. C-myc has been shown to regulate antitumor immune response through expression of immune-checkpoint molecules CD-47 and PD-L1 [26]. Using human tumor-derived cell lines and mouse models, Casey et al. showed that increased Myc activity correlated with expression of CD47 and PD-L1. Blocking Myc activity using shRNA or Bromodomain and Extra-Terminal domain protein (BET) inhibitors led to down regulation of CD47 and PDL1 expression, influx of innate and adaptive immune effector cells, followed by tumor regression [26]. Kortlever et al. in a subsequent study elegantly elucidated the mechanism by which Myc activation leads to increased tumor cell proliferation, macrophage infiltration, angiogenesis, and immune-evasion [27]. In a Kras-driven murine lung adenocarcinoma model, activation of Myc led to CCL9-mediated recruitment of PD-L1 expressing macrophages, PD-L1-dependent exclusion of T and B cells, and IL-23-mediated exclusion of NK cells. Inactivation of Myc in established tumors reversed all the stromal and immune changes with recruitment of T, B, and NK cells leading to apoptosis and regression of tumors to an indolent state. In this model, PD-L1 expression was entirely limited to tumor infiltrating macrophages and not the tumor cells. These findings strongly suggest MYC as a central regulator of global tumor-immune response. MYC-dependent tumors may be sensitive to immunotherapeutic agents and MYC expression may be a good biomarker for such sensitivity [19]. In addition to these, BET inhibitors appear to target MYC dependence in various malignancies by downregulating transcription of MYC, MYC-dependent target genes, and CD274 (encoding PD-L1) [28, 29], and their action was synergistic when used in combination with PD-L1 blockers [30, 31].

## 4. Conclusion

MYC-driven SCLC may be sensitive to AKIs, checkpoint blockers, and BET inhibitors. A significant proportion of SCLC tumors have amplification or overexpression of MYC family of genes. Prior clinical trials using agents targeting MYC in SCLC yielded modest results in unselected cases. Stratifying patients based on their tumor vulnerabilities using genomic biomarkers, and usage of corresponding targeted therapies, possibly in combination with existing treatment strategies is likely to produce better results in SCLC, who currently have extremely poor prognosis beyond first line chemotherapy.
